# Identification and sequence analyses of novel lipase encoding novel thermophillic bacilli isolated from Armenian geothermal springs

**DOI:** 10.1186/s12866-017-1016-4

**Published:** 2017-05-02

**Authors:** Grigor Shahinyan, Armine Margaryan, Hovik Panosyan, Armen Trchounian

**Affiliations:** 1 Research Institute of Biology, Yerevan State University, 0025 Yerevan, Armenia; 2 Department of Biochemistry, Microbiology and Biotechnology, Faculty of Biology, Yerevan State University, 0025 Yerevan, Armenia

**Keywords:** Geothermal springs, Thermophilic bacilli, Lipase, Esterase, GDSL family lipase

## Abstract

**Background:**

Among the huge diversity of thermophilic bacteria mainly bacilli have been reported as active thermostable lipase producers. Geothermal springs serve as the main source for isolation of thermostable lipase producing bacilli. Thermostable lipolytic enzymes, functioning in the harsh conditions, have promising applications in processing of organic chemicals, detergent formulation, synthesis of biosurfactants, pharmaceutical processing etc.

**Results:**

In order to study the distribution of lipase-producing thermophilic bacilli and their specific lipase protein primary structures, three lipase producers from different genera were isolated from mesothermal (27.5–70 °C) springs distributed on the territory of Armenia and Nagorno Karabakh. Based on phenotypic characteristics and 16S rRNA gene sequencing the isolates were identified as *Geobacillus* sp., *Bacillus licheniformis* and *Anoxibacillus flavithermus* strains. The lipase genes of isolates were sequenced by using initially designed primer sets. Multiple alignments generated from primary structures of the lipase proteins and annotated lipase protein sequences, conserved regions analysis and amino acid composition have illustrated the similarity (98–99%) of the lipases with true lipases (family I) and GDSL esterase family (family II). A conserved sequence block that determines the thermostability has been identified in the multiple alignments of the lipase proteins.

**Conclusions:**

The results are spreading light on the lipase producing bacilli distribution in geothermal springs in Armenia and Nagorno Karabakh. Newly isolated bacilli strains could be prospective source for thermostable lipases and their genes.

## Background

Thermophilic microorganisms are gaining big industrial and biotechnological interest due to their enzymes (thermozymes) remaining active in harsh industrial processes [1]. The industrial demand for the thermozymes continues to stimulate the search for novel thermophilic microorganisms from various unexplored regions of the Earth, such as deep-sea hydrothermal vents, terrestrial geothermal springs and mudpots [2].

One of the important groups of biotechnologically relevant enzymes are lipases (EC 3.1.1.3 - triacylglycerol hydrolases), which have found large applications in food, diary, detergent, and pharmaceutical industries [3, 4]. Lipases catalyse the hydrolysis of ester bonds of triacylglycerol at the interface between an insoluble substrate and water. In non-aqueous media these reactions are reversed due to a hydrophobic domain (lid), covering the active site of the lipase. The three-dimensional structures of lipases have shown their association with the α/β hydrolase family which contain terminal α-helices and a central β-sheet including the active Ser placed in a loop termed the catalytic elbow [5, 6]. Most of α/β hydrolases contain consensus sequence Gly-X-Ser-X-Gly around the active site serine, with a catalytic triad (Ser-Asp-His).

Lipase coding genes have been reported in wide range of microorganisms, however lipases derived from thermophiles have privileges compared to the mesophilic lipases due to their unique attributes [7]. Thermophilic bacilli are the natural source of many thermostable enzymes, among them the thermostable lipases which have received great attention in both structural studies and industrial applications as they show high stability at elevated temperatures and in organic solvents [8]. However, only a small numbers of lipase producing thermophilic microbes have been reported in the last decade. Thus, the screening for new thermostable lipase producers has become a very important task [3].

Thermophilic microorganisms appear in a big list of taxonomic groups and are located on different phylogenetic distances throughout the taxonomic trees of microorganisms [3, 9]. Among the huge diversity of thermophilic bacteria mainly bacilli have been reported as active thermostable lipase producers (1, 9–11). Number of thermophilic bacilli species belonging to the genera *Bacillus, Geobacillus* and *Anoxybacillus* have been isolated from different geotherms and reported as thermostable lipase producers [10–18].

It is shown recently that Armenian geothermal springs harboured thermophilic microbes from different taxonomic groups including thermophilic endospore-forming bacteria [19–21]. Despite this progress very little is known about the diversity of lipase producers thriving in Armenian geothermal springs.

The present study focuses on isolation and identification lipase-producing thermophilic bacilli from geothermal springs of Armenia (Akhourik and Tatev) and Nagorno-Karabakh (Karvachar) as well as comparative analysis of their lipase encoding genes’ sequences.

## Methods

### Sampling

Water temperature, pH, and conductivity were measured in situ during the sampling using a portable combined pH/EC/TDS/Temperature tester (HANNA HI98129/HI98130).

The geothermal spring in Akhourik is located in northwest Armenia at N 40° 44′ 34,04′′, E 43° 46′ 53,95′′, 1490 m above sea level, with a temperature of ≈ 30 °C, pH 6.5 and a conductivity 2490 μS/sm (Fig. 1.1). The geothermal spring in Tatev is located in southeast Armenia within the Syunik region at N 39°23.765′′, E 46°15. 482′′ with a temperature of ≈ 27.5 °C, pH 6.0 and a conductivity 1920 μS/cm (Fig. 1.2). The spring in Karvachar is located in north Nagorno-Karabakh at N 40°17.417′′, E 46°27.500′′ with a temperature of ≈ 70 °C, pH 7.3 and a conductivity 4600 μS/cm (Fig. 1.3).

**Fig. 1:**
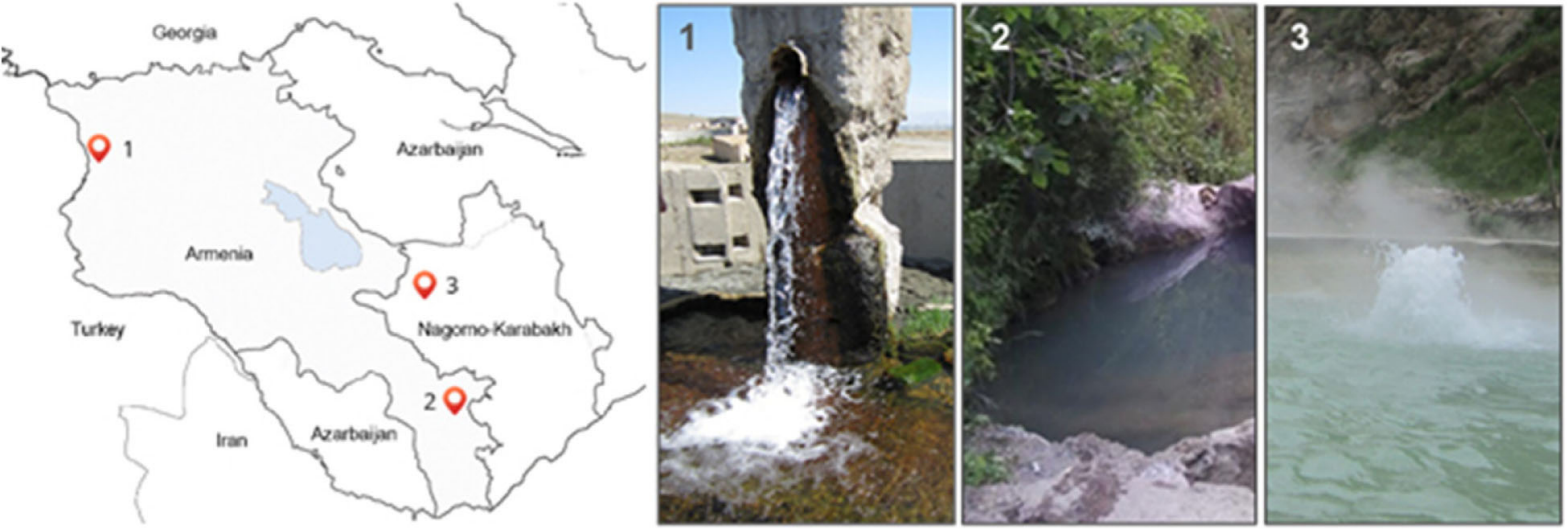
Location of study sites. Maps of Armenia and Nagorno Karabakh showing the locations of studied geothermal springs with red marks. Close up photograph of 1. Akhourik geothermal spring. 2. Tatev geothermal spring and 3. Karvachar geothermal spring. The source of the map: http://map-caucasus.com/

Sediment samples were aseptically collected from the outlet of the spring using sterile glass flasks and were maintained on ice until processing.

### Isolation and selection of lipase producing bacilli

To isolate thermophile bacilli 1.0 g of sediment samples was suspended in 10 ml of sterile water and mixed for 1 min. Supernatants were transferred to a glass tube with a screw cup and pasteurized at 80 °C for 10 min in a water bath. 1.0 ml aliquots were placed on enrichment medium and incubated with shaking (150 rpm) overnight at 55 and 65 °C. Enrichment medium had following composition: 0.6% nutrient broth (NB) and 1% Tween 80 or Olive oil as a substrate. Then 0.5 ml aliquots of appropriate dilution were placed on enrichment medium containing 1.5% agar and incubated overnight at 55 and 65 °C. All colonies obtained on plates were picked and purified by streaking on the same medium at least three times. The purified microbial isolates were screened for lipolytic activity on agar plates with lipoidal emulsion containing 6% olive oil, 0.05% Tween 80, 0.02% Rhodamine-B (Alken-Murray). Lipase producing isolates were screened on spread plates after incubation for 48 h at 55 and 65 °C. Isolates’ colonies exhibiting orange fluorescence zones under UV light at 312 nm were considered as active lipase producers [22].

### Phenotypic characteristics of the isolates

Microbial colonies were described by colour, size, shape, surface and margins on NB agar. The cell morphology, and motility, spore’s form and location were determined by light microscope (Motic 10, Hong Kong).

The temperature range for growth was determined after incubation of isolates at temperature from 25 to 75 °C with 10 °C intervals. The pH dependence of growth was tested at pH range from 4 to 11. The anaerobic growth, catalase and oxidase activity, reduction of nitrate to nitrite, Voges-Proskauer reaction, formation of dihydroxyacetone and indole were determined according to [23]. The utilization of citrate and different substrates as carbon sources (D-glucose, D-xylose and D-mannitol) was determined using the Simmon’s and Hugh Leyfsona’s medium, correspondingly [23]. The casein and starch hydrolyses were tested by streak glass plate technique. The ability to produce gelatinase was determined using the nutrient gelatine stab method [24].

### 16S rRNA gene (rDNA) analysis

Extraction of genomic DNA from pure cultures was carried out following to bacterial genomic DNA isolation using CTAB method [25]. Extracted DNA was used as template for amplification of the 27–1492 regions of 16S rDNA sequences by PCR. The “universal” oligonucleotide primers 16S F (5′-GAGTTTGATCCTGGCTCAG-3′) and 16S R (5′-GAAAGGAGGTGATCCAGCC-3′) were used for amplification of bacterial 16S rDNA [26]. PCR mixture with the final volume of 50 μl containing ≥100 ng DNA as a template, 10 μl 5xOne*Taq* Standard Reaction Buffer, 0.25 μl One*Taq* DNA Polymerase (BioLabs, New England), 1 μl 10 mM dNTPs, 0.5 μM of each primer. The cycling conditions: initial denaturation at 95 °C for 5 min followed by 30 cycles of denaturation at (1 min at 95 °C), primer annealing (40 s at 54 °C) and elongation (1 min at 68 °C), with final elongation step (10 min at 68 °C). The reactions were subsequently cooled to 4 °C. The PCR product was analysed on 1.0% agarose gel electrophoresis. PCR products were purified with GenElute™ PCR Clean-up Kit (Sigma).

### PCR amplification of lipase encoding genes

Isolated genomic DNA was used as a template amplification of lipase encoding genes. Primers for the amplification of the lipase encoding genes were designed based on appropriate gene sequences retrieved from NCBI GeneBank using Primer3 (v.0.4.0) and NCBI Primer-BLAST web tools (http://frodo.wi.mit.edu/; http://www.ncbi.nlm.nih.gov/tools/primer-blast). Oligonucleotide sequences of designed primers are given in Table 1. The amplification mixture with the final volume of 50 μl containing ≥100 ng DNA, 10 μl 5xOne*Taq* Standard Reaction Buffer, 0.25 μl One*Taq* DNA Polymerase (BioLabs, New England), 1 μl 10 mM dNTPs, 0.5 μM of each primer.

**Table 1:** Primers used for lipase encoding gene PCR amplification

Gene name	Forward Primer	Reverse Primer	Tm (°C)	Product size (bp)
Lipase encoding gene in *Anoxybacillus* (*lipA*)	ATTCTCCTATCCATTCATCATATT	TTTCTTCTTCTGTTCCTTTGC	54	733
Lipase encoding gene in *Bacillus* (*lipB*)	TATGCGTCGTCATTCATTTT	GGTCAGGCCATCTTTAATCA	54	601
Lipase encoding gene in *Geobacillus* (*lipC*)	GGTTGTGTTGCTCGGATTA	CAAACTCGCCAGTTGCTC	56	1222

Amplification was performed under conditions of an initial denaturation at 94 °C for 3 min followed by 30 cycles of denaturation at 94 °C for 1 min, annealing at 54 (or 56 °C) for 30 s, extension at 68 °C for 1 min, with final extension at 68 °C for 10 min. The reactions were subsequently cooled to 4 °C. The PCR product was analyzed on 1.0% agarose gel electrophoresis.

### Sequencing and phylogeny

Sequencing of bacterial 16S rDNA amplicons and lipase encoding genes were performed on ABI PRISM capillary sequencer according to the protocol of the ABI Prism BigDye Terminator kit (Perkin Elmer) using above mentioned primers. The presence of the chimeric sequences was determined using the DECIPHER web tool (http://decipher.cee.wisc.edu/FindChimeras.html) [27]. Raw data of DNA sequences was analysed with Chromas and BioEdit software. The 16S rDNA and lipase encoding genes sequences were aligned and compared with other 16S rRNA gene sequences in GenBank by using the NCBI Basic Local Alignment Search Tools, nucleotide (BLASTn) program (http://www.ncbi.nlm.nih.gov/BLAST/). Alignment for phylogenetic analysis of 16S rDNA was made by using ClustalW [28]. Phylogenetic tree was constructed using the neighbour joining method with MEGA 6.06 software [29]. Bootstrapping analysis for 1000 replicates was performed to estimate the confidence of tree topologies [30].

Prediction of signal peptide amino acid sequences based on lipase gene sequences was performed by EMBOSS Transeq tool in EMBL-EBI web service (http://www.ebi.ac.uk/Tools/st/). The lipase Engineering Database was searched using the BLASTP program. Multiple sequence alignments were constructed using PROMALS3D multiple sequence and structure alignment online server [31]. To determine the evolutionary relationship of bacterial lipases with other lipases, phylogenetic analysis was performed with the amino acid sequences of bacterial lipases and 12 other lipases (see Table 2) obtained from the NCBI database using neighbour-joining methods with 1000 bootstrap replications performed by MEGA 6.06 software [29].

**Table 2:** The numbering of bacterial lipases obtained from NCBI database used in silico analyses

Protein ID	Accession number	Protein name	Bacterial strain	Isolation sours	Reference
Lipase 1	WP_035066469	lipase	*A. gonensis*	Gonen and Diyadin hot springs, Turkey	[32]
Lipase 2	WP_043966778	lipase	*A. thermarum*	Euganean hot springs, Italy	[33]
Lipase 3	WP_012575053	lipase	*A. flavithermus*	Waste water drain at the Wairakei geothermal power station, New Zealand	[34]
Lipase 4	WP_055441552	GDSL family lipase	*A. suryakundensis*	Hot Spring in Jharkhand, India	[35]
Lipase 5	AHJ58872	lipase	*B. licheniformis*	NP	NP
Esterase 6	WP_003183220	esterase	*Bacillus* sp.	NP	NP
Esterase 7	WP_043054382	esterase	*B. paralicheniformis*	NP	NP
Esterase 8	WP_020452056	alkaliphilic lipase EstA	*B. subtilis*	NP	NP
Esterase 9	WP_029418313	esterase	*B. sonorensis*	Sonoran Desert soil, USA	[36]
Lipase 10	ACN79581	thermostable lipase	*Geobacillus* sp.	Compost of local area of Taiwan	[37]
Lipase 11	KFX34290	lipase	*G. icigianus*	Hot Springs in the Valley of Geysers, Kamchatka, Russian Federation	[38]
Lipase 12	AFU07645	lipase	*Geobacillus* sp.	Botanischer Garten, University of Hamburg, Germany	[39]

*Abbreviation*: *NP* not published

### Nucleotide sequence accession numbers

The 16S rRNA gene, as well as lipase encoding gene sequences described in this report have been submitted to GenBank under the following accession numbers: KY203974 to KY203976 (for 16S rRNA genes) and KY213839 to KY213841 (for lipase encoding genes).

## Results

### Isolation and phenotypic characterisation

Collected three sediment samples were analysed to evaluate the lipase producing aerobic thermophilic endospore-forming bacterial abundance in Armenian geothermal springs. In total forty-one chemoorganotrophic thermophilic aerobic lipase-producer bacilli strains were isolated from Akhourik, Tatev and Karvachar geothermal springs. Three most active producers of which designated as Akhourik 107, Tatev 4 and Karvachar QB2 were selected and further characterized. All of the isolates were routinely maintained at 4 °C on NB agar slants.

Selected lipase-producing isolates were characterized by their phenotypic properties. On solid NB medium isolates differ by colony form, colour and surface, thus they form light yellow, white or creamy, circular, smooth or transparent colonies with 0.5–2 mm diameter. The isolates were Gram positive, endospore forming motile rods varying in length between 1.8 to 7.8 μm and in width between 0.5 to 2.0 μm (Table 3).

**Table 3:** Some phenotypic characteristics of the studied isolates

Phenotypic characteristics	*Akhurik 107*	*Tatev* *65–4*	*Karvachar* *QB2*	*B. subtilis ATCC6051*	*G. toebii SK-1*	*A. flavithermus DSM 2641*
*Cell size (*μm*)*						
*width*	1.0–2.0	1.2	1.0	0.7–0.8	0.5–0.9	0.8–0.85
*length*	4.0–5.0	4.25	1.8	2–3	2–3.5	2.3–7.1
Endospore						
form	Ellipsoidal	Ellipsoidal	Ellipsoidal	Ellipsoidal	Ellipsoidal	Ellipsoidal
location	Central	Sub-terminal	Terminal	Central	Sub-terminal to terminal	Terminal
Swell sporangia	−	+	+	−	+	+
Temperature range (optimum) (°C)	30–60 (55)	45–70 (65)	40–70 (60)	20–55 (28–30)	45–70 (60)	30–72 (60–65)
pH range (optimum)	5–10 (7–8)	6–9 (7)	7–11 (9–10)	5.5–8.5 (7)	6–9 (7.5)	5.5–9 (7)
NaCl range (%)	≤5	≤2.5	≤2	≤7	≤5	≤2.5
Voges-Proskauer test	+	−	−	+	+	+
Acid formation from						
D-glucose	+	−	−	+	+	+
Xylose	+	−	−	+	−	−
Mannitol	+	−	−	+	−	+
Nitrate reduction to nitrite	+	+	−	+	+	+
Hydrolysis of						
Starch	+	−	+	+	−	−
Gelatine	+	−	+	+	−	−
Casein	+	+	+	+	+	−
Utilization of citrate	−	+	−	+	−	ND
Formation of dihydroxyacetone	+	−	−	−	−	−

*Abbreviations*: *+* positive reaction, − negative reaction, *ND* not determined. The phenotyping characteristics of the type strains *B. subtilis* ATCC6051 [40], *G. toebii SK-1* [40, 41] and *A. flavithermus DSM 2641* [42, 43] were used as references.

All isolates were facultative anaerobic and have shown ability to grow in wide range of temperature and pH. The optimal growth temperature values of studied isolates varied from 55 °C – 65 °C. Isolate Akhourik 107 is also able to grow under 37 °C which clearly shows the strain to be thermotolerant. Bacterial isolates grow optimally at pH 7.0 except for Karvachar QB2, which grows optimally at pH 9.0.

The highest tolerance towards NaCl content in the media was measured for Akhurik 107 (5%). Other isolates have tolerated 2–2.5% of sodium chloride concentrations and have shown optimal growth in 0–3% NaCl content in media.

The strain Akhourik 107 was positive according to Voges-Proskauer test, while others were negative. The isolates of Akhourik 107 formed dihydroxyacetone and reduced nitrate to nitrite.

The isolates (except Tatev 4) hydrolysed starch and gelatine, the isolate Tatev 4 utilized citrate as a carbon source. A list of phenotypic characteristics of the isolates is displayed in the Table 2.Morphological, biochemical and physiological characteristics of isolates were showed their similarity to the *Bacillus* and related genera. Further molecular identification of bacterial isolates using 16S rDNA sequencing method was carried out in order to recover the phylogenetic relationship between the isolates.

### Phylogenetic analysis of the isolates

For further identification of the strains, genomic DNA was purified and 16S rDNA was amplified using 16SF and 16SR primers. The near full-length 16S rDNA nucleotide sequences were aligned against the 16S rRNA sequences in GenBank by using the NCBI BLASTn program. BLAST results for the isolates based on 16S rDNA sequences for identification of the closest relatives in the GenBank database are reported in Table 4.

**Table 4:** Closest phylogenetic affiliation of strains’ 16S rDNA based on BLAST comparison to the GenBank database

Isolate	Accession number in GenBank	Sequence length (bp)	Closest phylogenetic match and Accession numbers	Similarity %
Akhourik 107	KY203975	944	*Bacillus licheniformis* strain HT-Z71-B2 |KJ526873|	99
Tatev 4	KY203974	920	*Geobacillus* sp. C170 |FJ848022|	99
Karvachar QB2	KY203976	1443	*Anoxybacillus flavithermus* strain AK1 |KC503890|	99

**Fig. 2:**
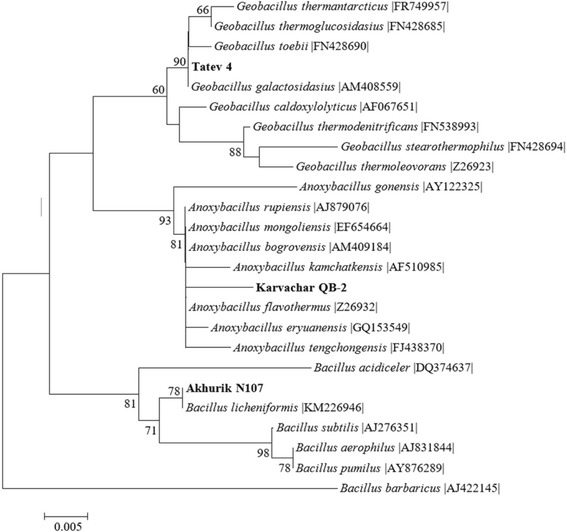
Phylogenetic tree obtained by distance matrix analysis showing the position of the strains Akhurik 107, Tatev 4 and Karvachar QB2. The tree was generated by the Neighbour-Joining method using MEGA 6.06 software. Bootstrap values per 100 bootstrap analysis are presented on the tree. Scale bar represents 0.005 substitutions per site

The phylogenetic tree based on sequences of isolates and reference strains of belonging to genera *Anoxybacillus, Bacillis* and *Geobacillus* is displayed in Fig. 2.

The phylogenetic tree confirms that isolate Tatev 4 constitute a part of the cluster within one of the thermophilic groups of bacilli (genus *Geobacillus*). Another thermophilic bacilli cluster contains isolate Karvachar QB2 with an affiliation of specie from genus *Anoxybacillus*. A separate cluster contains isolate Akhourik 107, which was affiliated with genera *Bacillus* (see Fig. 2).

The phylogenetic tree illustrates facts that were not visible on the closest affiliation analysis (BLAST) results. Even though the BLAST has shown the QB2 to be 99% similar to *Anoxybacillus flavithermus* strain AK1, the Neighbour-Joining method used has demonstrated that the QB2 is relatively further located on the tree in the closer with *Anoxybacillus flavithermus* strain AK1. In the case of Tatev 4 strain the BLAST has shown their similarity with *Geobacillus* sp., but in the phylogenetic tree it closely located with *G. galactosicus* in the same cluster.

Amplification of lipase genes by designed primer sets was successful for the *B. licheniformis* Akhourik 107, *Geobacillus* sp. Tatev 4 and *Anoxybacillus* sp. QB2 strains. The nucleotide sequences were translated to amino acid sequences using EMBOSS Transeq tool in EMBL-EBI web service. Alignment of various lipase/esterase from bacilli in GenBank revealed sequence similarity at protein level (Table 5). The pairwise identities of the protein sequences ranged from 98 to 99%.

**Table 5:** Closest phylogenetic affiliation of lipase protein sequences based on BLAST comparison to the GenBank database

Protein ID	Accession number in GenBank	Number of amino acids	Closest phylogenetic match and Accession	Similarity %
LipA	KY213839	242	lipase (*Anoxybacillus* sp. KU2–6(11)) |WP_035048205|	99
LipB	KY213840	202	MULTISPECIES: esterase (*Bacillus*) |WP_003183220|	99
LipG	KY213841	400	lipase (*Geobacillus* sp. GHH01) |WP_041470719|	98

**Fig. 3:**
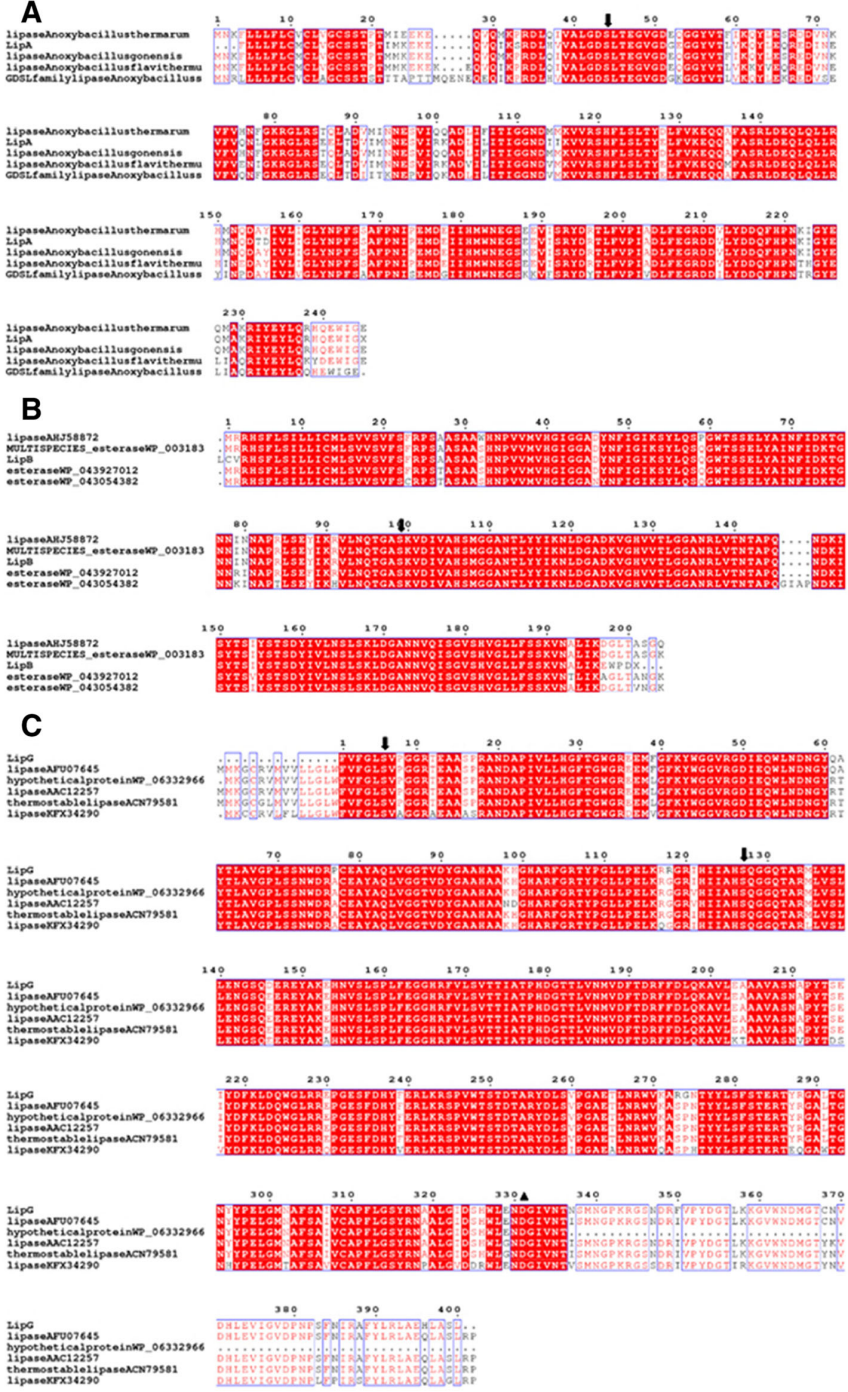
Multiple sequence alignment for *A. flavithermus* QB2 **a** *B. licheniformis* Akhurik 107 **b** *Geobacillus* sp. Tatev 4 **c** strains and closely related lipases/esterases. Protein sequences were aligned with CLUSTALX (2.0.11) and ESPrint (3.0). Identical residues were marked with red background and the highly-conserved residues were showed as red font. The catalytic triads are indicated with arrow. Тhe triangle is showed aspartic residues involving in the Ca^2+^-binding site

A multiple alignment was constructed to illustrate conserved and variable sites within the retrieved lipase subfamilies (Fig. 3). Conserved regions in all aligned lipases were found. Lipase of *Anoxybacillus* sp. QB2 (LipA) have conserved regions with the sequences of the lipase derived from *A. gonensis* (WP_035066469) and *A. thermarum* (WP_043966778). In the multiple sequence alignment of *Anoxybacillus* sp. QB2 lipase amino acid sequence of and closely related lipases display a GlyAsp-Ser-(Leu) (GDSL) motif [6] containing the active-site serine residue (Fig. 3a).

Lipase of *B. licheniformis* Akhourik 107 and *Geobacillus* sp. Tatev 4 have conserve regions with the esterase and thermostable lipases, correspondingly with active-site serine residue in the Asp-His-Ser catalytic triad (Fig. 3b, c) [6].

**Fig. 4:**
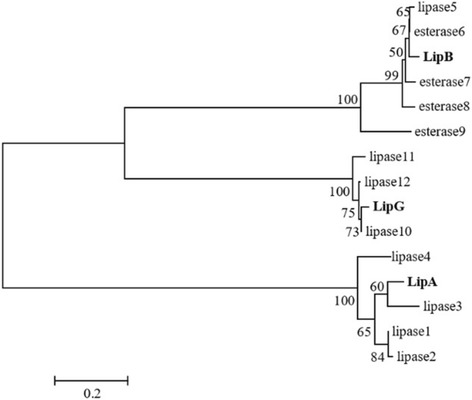
Phylogenetic analysis of lipase sequences (partial sequence, 200 amino acids). The data are illustrated as a neighbour-joining tree based on 12 lipase sequences from the NCBI Database

The phylogenetic tree constructed by the NJ method for the lipase family proteins is shown in Fig. 4.

Lipase of *Anoxybacillus* sp. QB2 (LipA) cluster loosely together with lipase derived from *Anoxybacillus flavithermus* WP_012575053. Lipase of *B. licheniformis* Akhourik 107 and *Geobacillus* sp. Tatev 4 cluster together with esterase and lipases derived from *Geobacillus* sp., correspondingly.

## Discussion

The geology of the region where Armenia and Nagorno-Karabakh are situated is complex, owing to accretion of terrains through plate-tectonic processes, and to ongoing tectonic activity and volcanism. A belt of quaternary volcanism led formation of the numerous low-temperature and medium hot springs in the territories of Armenia. The studies of the geothermal springs in the territory of Armenia indicated that compared with the upland some springs have much higher temperatures at deeper levels, where water temperature can reach up to 99 °C [44]. The diversity of microbes including endospore-forming bacteria have been studied in the Armenian geothermal springs [19–21]. However, the diversity of lipase producers thriving in Armenian geothermal springs is little known.

In order to fill in the gap of knowledge about the lipase producing bacilli found in the geotherms on the territory of Armenia and Nagorno Karabakh, 40 chemoorganotrophic bacilli strains were isolated from sediment samples of Akhurik, Tatev and Karvachar geothermal springs on the enrichment media containing Tween 80 or olive oil as a substrate. Three isolates were selected as potential lipase producers and were characterized. It is particularly interesting that the isolates from Tatev and Akhurik geothermal springs are optimally growing at temperature of 65 and 55 °C, correspondingly (see Table 3), even though the native geothermal springs temperature in the outlet is around 28–30 °C. Geological studies of the region have shown that the wells are characterized by relatively low temperatures (25° to 43 °C), but have high artesian flow rates (from 10 l/s up to a reported 102 l/s). The hotter wells (40 °C or greater) range from 700 m to 1150 m in depth [44]. The comparison of the optimum growth temperature of the isolates to the water temperature of geothermal spring in the deep suggests that most of the detected microorganisms are likely able to grow at reservoir temperatures and, therefore, should not be regarded as contaminants.

The properties of the isolates to utilize several mono- and disaccharides as carbon sources (production of acid, but no gas), digestion of gelatine, starch and casein showed some differences compared with reference strains (see Table 3). The isolate Akhurik 107 is not able to utilize citrate, but can grow anaerobically and form dihydroxyacetone which is not described for *B. subtilis* ATCC6051 strain. The isolate Tatev 4 compared with *G. toebii* SK-1 strain is not able to utilize glucose as carbon source, although can metabolize citrate (see Table 3). The phenotype of the isolate Karvachar QB2 differed from the phenotype of the *A. flavithermus* DSM 2641 type strain showing high hydrolase activities, while not being able to utilise the studied sugars as carbon sources (see Table 3).

The BLAST analysis of 16S rDNA sequences revealed the close relationship of Akhourik 107 to *B. licheniformis* (99%) (see Table 4) and the position of the isolate Akhurik 107 in the phylogenetic tree next to *B. licheniformis* |KM226946| strain (see Fig. 2) clearly indicate that the studied isolate is one of the *B. licheniformis* species. The BLAST analysis of 16S rDNA sequences of Tatev 4 and Karvachar QB2 have shown the similarities with the *Geobacillus* sp. C170 (99%) and *A. flavithermus* AK1 (99%). The phylogenetic analysis has shown the close relationship between the isolate Tatev 4 and *G. galactosidasius* |AM408559|. More detailed studies are necessary to confirm taxonomic affiliation of the isolates.

Number of thermophilic bacilli species belonging to the genera *Bacillus, Geobacillus* and *Anoxybacillus* have been isolated from different geothermal springs and reported as thermostable lipase producers [10–13, 15–18]. Previous studies report that *B. thermoamylovorans* CH6B lipolytic enzyme-producing thermophilic microorganism have been isolated from a hot spring in Galicia (North Western Spain) [37]. Olusesan A. T. et al. [18] isolated *A. kamchatkensis* starin KW 12 from Malaysian hot spring and characterized as lipase producers. It seems that the bacilli genera mentioned are the dominating lipase-producing bacterial groups found in different hot springs. Therefore, the potential of *Bacillus, Geobacillus, Anoxybacillus* and *Brevibacillus* species, as candidates for commercial production of thermostable lipases should be considered.

In the presented work, the lipase gene specific primer sets were constructed to determine the lipase genes in the genome of the newly isolated lipase active bacilli of *Anoxybacillus, Bacillus* and *Geobacillus* genera. Based on the gene sequences, the lipase amino acid sequences were constructed and analysed. The putative lipases of *A. flavithermus* Karvachar QB2, *B.licheniformis* Akhurik 107 and *Geobacillus* sp. Tatev 4 consist of 242, 202 and 400 amino acid residues correspondingly. The deduced amino acid sequences of LipA, LipB and LipG proteins showed the highest similarity with lipases from *Anoxybacillus* sp. KU2–6(11) (99%), esterase from *Bacillus* sp. (99%), and lipase from *Geobacillus* sp. GHH01 (98%) respectively (see Table 5). The high sequence similarity has shown the close relationship between the proteins and made it easy to consider the association of the proteins to the same subfamilies [45].

The most similar sequences with identity scores between 99% and 96% were used for the multiple alignment analysis. Figure. 3 shows the LipA, LipB and LipG protein sequences alignments with related carboxylases. Multiple alignments generated for LipA and close related lipase protein sequences displayed conserved motifs containing the active site serine residue (see Fig. 3a). The revealed conserved sequence block is typical for GDSL esterase family (family II) (4), which is reportedly characterized to be highly thermostable and active within 6–11 pH range [46].

Various *Bacillus* lipases are known to have the first gliycine residue replaced with alanine in the conserved pentapeptide: Ala-X-Ser-X-Gly as a common feature (4). However, the lipase form *B. licheniformise* Akhurik 107 does not exhibit the typical pentapeptide and rather display a Gly-Asp-Ser motif, characterising the enzymes grouped in family II.

Even though LipA and LipB are close to the esterases in the phylogenetic tree, these proteins are located in separate clusters in Fig. 4, which indicates the purposed structural/functional differences between two lipolytic enzymes.

The multiple alignments of LipG protein and closely related lipases contain blocks of conserved sequences Intrinsic for true lipases from family I (4). LipG protein is characterised with the presence of aspartic residues involved in Ca^2+^- binding site (Fig. 3c).

The result of phylogenetic analysis of the lipase proteins derived from the isolated *Bacillus*, *Anoxybacillus* and *Geobacillus* strains and the most similar sequences with identity scores between 96% and 99% in BLAST analysis showed that LipA is situated in the same cluster with the lipase 3. Lipase 3 is a lipase obtained from *A. flavithermus* WK1 strain, which has been isolated from the waste water drain it the Wairakei geothermal power station, New Zealand [34]. LipG and LipB are forming a cluster with lipase 10 and lipase 5 to 6, correspondingly. Lipase 10 is a thermostable lipase protein synthesised by *Geobacillus* sp. strain NTU 03 isolated from compost of on the area of Taiwan [37]. Lipase 5 and 6 are esterases which are obtained from *Bacillus licheniformis* (see Table 2). Considering the obtained results, a conclusion was made that independent from the environment, whether it is a geothermal spring in Armenia, in New Zealand or compost in Taiwan, the evolution of the thermostability of the lipases has a very similar pattern.

The studied lipolytic proteins differ with their structure and therefore by functions and should be further analysed as prospects for different biotechnological applications.

## Conclusions

The results widely extended the previously acquired information regarding the thermophilic lipase producing bacilli diversity of geothermal springs in Armenia and Nagorno Karabakh while showing the importance of further investigation of the microbial community structure in Armenian geothermal springs to discover and isolate new thermophilic lipase producing species. The novel thermophilic bacilli isolates serve as potential source for thermostable lipases and have promising application in biotechnology and industry. Obviously, the geothermal springs contain a strong potential for further exploration of new thermozymes producing bacteria.

## Abbreviations

BLAST: Basic Local Alignment Search Tools
CTAB: Cetyl trimethylammonium bromide
DNA: Deoxyribonucleic acid
lipA: Lipase encoding gene in *Anoxybacillus*
lipB: Lipase encoding gene in *Bacillus*
lipG: Lipase encoding gene in *Geobacillus*
NB: Nutrient broth
PCR: Polymerase chain reaction
rRNA: Ribosomal ribonucleic acid
